# Integrated analysis of a competing endogenous RNA network reveals key lncRNAs as potential prognostic biomarkers for human bladder cancer

**DOI:** 10.1097/MD.0000000000011887

**Published:** 2018-08-21

**Authors:** Naiqiang Zhu, Jingyi Hou, Yuanhao Wu, Jinxin Liu, Geng Li, Wenjia Zhao, Guiyun Ma, Bin Chen, Youxin Song

**Affiliations:** a Affiliated Hospital of Chengde Medical College; b Hebei Key Laboratory of Study and Exploitation of Chinese Medicine, Chengde Medical College, Chengde; c First Teaching Hospital of Tianjin University of Traditional Chinese Medicine, Tianjin; d China-Japan Friendship Hospital, Beijing; e Tianjin University of Traditional Chinese Medicine, Tianjin, China.

**Keywords:** competing endogenous RNA, differentially expressed RNAs, human bladder cancer, long-noncoding RNA

## Abstract

Human bladder cancer (BCa) is one of the most commonly diagnosed malignancies worldwide. It has high recurrence rates and low-grade malignancy, thus representing an important public health concern. An increasing number of studies suggest that long-noncoding RNAs (lncRNAs) play important roles in various biological processes and disease pathologies, including cancer.

We analyzed the expression profiles of lncRNA, miRNA, and mRNA, along with the clinical information of BCa patients collected from the Cancer Genome Atlas database to identify lncRNA biomarkers for prognosis. We also constructed an lncRNA–miRNA–mRNA global triple network (competitive endogenous RNA network) by bioinformational approach.

This BCa lncRNA–miRNA–mRNA network consisted of 23 miRNA nodes, 52 mRNA nodes, 59 lncRNA nodes, and 365 edges. Subsequent gene ontology (GO) and pathway analyses were performed using BinGO for Cytoscape and Database for Annotation, Visualization, and Integration Discovery, respectively, highlighting important GO terms and pathways that were enriched in the network. Subnetworks were created using 3 key lncRNAs (*MAGI2-AS3*, *ADAMTS9-AS2*, and *LINC00330*), revealing associations with BCa-linked mRNAs and miRNAs. Finally, an analysis of significantly differentiating RNAs found 6 DElncRNAs (*AC112721.1*, *ADAMTS9-AS1*, *ADAMTS9-AS2*, *HCG22*, *MYO16-AS1*, and *SACS-AS1*), 1 DEmiRNA (*miRNA-195*), and 6 DEmRNAs (*CCNB1*, *FAM129A*, *MAP1B*, *TMEM100*, *AIFM3*, and *HOXB5*) that correlated with BCa patient survival.

Our results provide a novel perspective from which to study the lncRNA-related ceRNA network in BCa, contributing to the development of future diagnostic biomarkers and therapeutic targets.

## Abstract 2

Supplemental Digital Content is available in the text

## 1 Introduction

Human bladder cancer (BCa) is one of the most common malignancies worldwide, with high morbidity and mortality.^[1]^ BCa encompasses a wide spectrum of disease, ranging from superficial, well-differentiated carcinomas that do not affect survival, to highly aggressive tumors with poor prognosis.^[2,3]^ In 2017, there were approximately 79,030 new cases diagnosed in the United States (60,490 males and 18,540 females), leading to 16,870 deaths (12,240 males and 4630 females).^[4]^ Although BCa is linked to several universal genetic changes, identifying the mechanisms that underlie cancer progression remains challenging due to complicated disease processes and numerous molecular interactions. Therefore, the identification of potential biomarkers and novel targets for prognosis, diagnosis, and treatment is urgently needed.

Recently, the role that long-noncoding RNAs (lncRNAs) may play in cancer progression has received increased attention. These are nonprotein coding transcripts longer than 200 nucleotides^[5]^ that are broadly distributed throughout the genome.^[6–9]^ Previous studies have demonstrated that lncRNAs are involved in modulating gene expression at the transcriptional, post-transcriptional, and epigenetic levels.^[10]^ Evidence also suggests that lncRNAs contribute to the control of a variety of biological processes (BP), including the maintenance of genome integrity, stem cell pluripotency, cell differentiation, genomic imprinting, and X inactivation.^[11–13]^ As such, lncRNAs are thought to associate with the pathogenesis of many different cancers, including hepatocellular liver cancer,^[14]^ esophageal squamous cell carcinoma,^[15]^ colorectal cancer,^[16]^ renal cell carcinoma,^[17]^ gastric cancer,^[18]^ and prostate cancer.^[19]^ These studies indicate that lncRNAs could potentially serve as diagnostic or prognostic markers for human cancer, including BCa. However, the exact functions of most lncRNAs, including any putative role in BCa, are unclear and further study is urgently required.

In order to better understand how noncoding RNAs (ncRNAs) may be involved in BP and pathogenesis, a competitive endogenous RNA (ceRNA) network hypothesis has been proposed.^[20]^ This framework aims to describe how 3 types of RNA transcript (lncRNA, miRNA, and mRNA) interact to regulate transcription, creating a new fundamental “language” that describes transcriptional control by miRNA binding sites and miRNA response elements. A key hypothesis of ceRNA theory is that miRNAs play an important role in the ceRNA network by binding mRNA, inhibiting mRNA expression. LncRNAs also affect transcription by competing with miRNAs, subsequently affecting expression of the target mRNA.^[21]^ Research has also shown that ceRNA and related theories may provide an important new tool to advance tumor diagnoses and treatment options. For example, Sumazin et al^[22]^ systematically investigated an mRNA-related ceRNA network in glioblastoma cells, confirming that these ceRNA interactions mediate the crosstalk between oncogenic pathways. Aiding this research is several well-established RNA databases that provide useful information to understand ncRNA-mediated ceRNA regulatory mechanisms. These include the long-noncoding RNA-associated diseases (LncRNADisease) database,^[23]^ the Human miRNA Disease Database,^[24]^ and database of Differentially Expressed MiRNAs in human Cancers dbDEMC.^[25]^ There are also several miRNA-target interactions databases, including miRcode^[26]^ and miRanda,^[27–29]^ and the ceRNA-specific long-noncoding competing endogenous database (lnCeDB).^[30]^

To establish if lncRNAs have a role in the progression of BCa, our study constructed a global triple RNA network based on ceRNA theory using data from the Cancer Genome Atlas (TCGA). Gene ontology (GO) and pathway analyses were performed using the BinGO plug-in for Cytoscape and the Database for Annotation, Visualization, and Integration Discovery (DAVID), respectively, to reveal any associations between mRNAs in the network and BCa. Next, important hub lncRNAs were identified in the lncRNA–miRNA–mRNA network and new subnetworks formulated that centered on these lncRNAs. Further GO and pathway analysis of these subnetworks revealed several important processes and miRNAs that link these lncRNAs to BCa. These data provide valuable insights into the molecular progression of BCa and will contribute to identify potential mechanisms of pathogenesis. This will improve the diagnosis and prognosis of BCa, in addition to aiding the identification of putative drug targets.

## 2 Materials and methods

### 2.1 Data collection and preprocessing

A total of 418 patients with BCa were enrolled in our comprehensive integrated analysis. Data were downloaded from the TCGA database (http://tcga-data.nci.nih.gov/) using the Data Transfer Tool provided with GDC Apps. For the study, level 3 mRNASeq gene expression data, miRNASeq data, and clinical information of patients were downloaded (http://tcga-data.nci.nih.gov/). Sequencing data were collected using Illumina HiSeq RNASeq and Illumina HiSeq miRNASeq platforms (Illumina, San Diego, CA) and the study was performed in line with the publication guidelines provided by TCGA (http://cancergenome.nih.gov/publicaitons/publicationguidelines). Ethical approval was not necessary in our study because the expression profiles were downloaded from the public database and no new experiments in patients or animals were performed.

### 2.2 Screening of differentially expressed genes

EdgeR (http://bioconductor.org/packages/release/bioc/html/edgeR.html)^[31]^ was used to screen differentially expressed lncRNAs (DElncRNAs), miRNAs (DEmiRNAs), and mRNAs (DEmRNAs) from the dataset by comparing normal and BCa groups. *P* values were calculated using post hoc tests using a significance threshold of *P* < .01 and |fold change (FC)| > 2.0. Hierarchical clustering was visualized using the gplots package of R (http://cran.r-project.org/web/packages/gplots/index.html).^[32]^

### 2.3 Competing endogenous RNA network analysis

To investigate the potential roles of lncRNAs within the mediated ceRNA network, a coexpression network of DEmRNAs, DElncRNAs, and DEmiRNAs was built using Cytoscape v 3.5.1 software.^[33]^ miRNA-targeted mRNAs were retrieved from miRTarBase (http://mirtarbase.mbc.nctu.edu.tw/). Each miRNA–mRNA pair used was preciously experimentally validated by reporter assay and at least 2 of the following methods; qRT-PCR, western blotting, microarray, and next-generation sequencing experiments in miRTarBase. In addition, lncRNA–miRNA interactions were constructed based on miRcode (http://www.microde.org/).^[26]^

### 2.4 GO and pathway analysis

The GO analysis database (http://geneontology.org) was used to annotates genes and gene products to identify associating biological attributes in the transcriptome and high-throughput genome data.^[34,35]^ Similarly, the Kyoto Encyclopedia of Genes and Genomes (KEGG) knowledge database (http://www.kegg.jp/) was used for the systematic analysis of gene functions, linking genomic information with higher-order functional information.^[36]^ To evaluate pathways and BP that were DE in the ceRNA network, the DAVID^[37,38]^ (http://david.abcc.ncifcrf.gov/) and the BinGO^[39]^ plug-in for Cytoscape were used for functional enrichment analysis. KEGG pathways and GO BP were highlighted at a significance threshold of *P* < .05^[40]^ and a −log 10 (*P*) denoted enrichment scores with significant pathway correlations.

### 2.5 Construction of key lncRNA–miRNA–mRNA subnetworks

Each downloaded lncRNA, and its linked miRNAs and mRNAs in the ceRNA network, were extracted and used to construct new subnetworks using Cytoscape software. In the study, degree centrality (a fundamental parameter in network theory) was adopted to evaluate each node in order to identify key lncRNAs in the subnetwork. Degree centrality was defined as the number of adjacent links for the node and was determined from the number of interactions that connected each RNA to its neighbors. The degree centrality method was calculated using the CytoHubba^[41]^ plug-in for Cytoscape. Further analysis involved identifying the GO and pathway annotations for each of the key lncRNAs by establishing their mRNA neighbors in the lncRNA–miRNA–mRNA subnetwork. GO interaction networks were then reconstructed using the BinGO plug-in for Cytoscape.

### 2.6 Survival analysis

To identify any prognostic DEmRNA, DEmiRNA, or DElncRNA signatures, clinical data from patients with BCa were collected from the TCGA. Survival curves for each sample with DElncRNAs, DEmiRNAs, and DEmRNAs were creating using the “survival” R package. Univariate survival was estimated using a Kaplan–Meier univariate survival method.^[42]^*P* < .01 was considered significant.

## 3 Results

### 3.1 Identifying DEmRNAs, DEmiRNAs, and DElncRNAs in BCa samples

**Figure 1:**
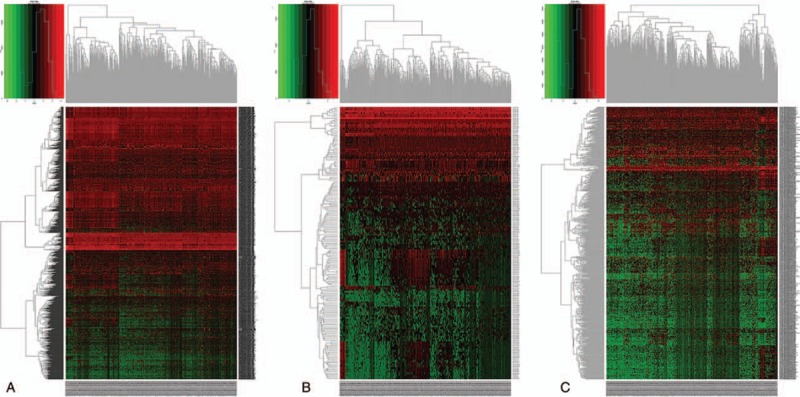
Heatmap of differentially expressed (DE) RNAs comparing patients with bladder cancer to controls. (A) DEmRNAs, (B) DEmiRNAs, and (C) DElncRNAs are shown. The left vertical axis indicates the clusters of differentially expressed RNAs, whereas the right vertical axis indicates RNA names. Red represents up-regulated RNAs and green represents down-regulated RNAs.

The RNA expression profiles of patients with BCa and corresponding clinical information were downloaded from the TCGA database using the Data Transfer Tool. We then used EdgeR to identify significantly DEmRNAs, DEmiRNAs, and DElncRNAs between BCa and normal samples (DEmRNAs, DEmiRNAs, and DElncRNAs). This identified a total of 1819 DEmRNAs, 157 DEmiRNAs, and 666 DElncRNAs. More specifically, there were 1030 (56.2%) up-regulated and 789 (43.37%) down-regulated DEmRNAs (Table S1), 131 (83.4%) up-regulated and 26 (16.5%) down-regulated DEmiRNAs (Table S2), and 246 (36.9%) down-regulated DElncRNAs and 420 (63.1%) up-regulated DElncRNAs identified (Table S3). A heat map demonstrating the complete linkage clustering of DEmRNAs, DEmiRNAs, and DElncRNAs is shown in Fig. 1.

### 3.2 Construction of the ceRNA network

**Figure 2:**
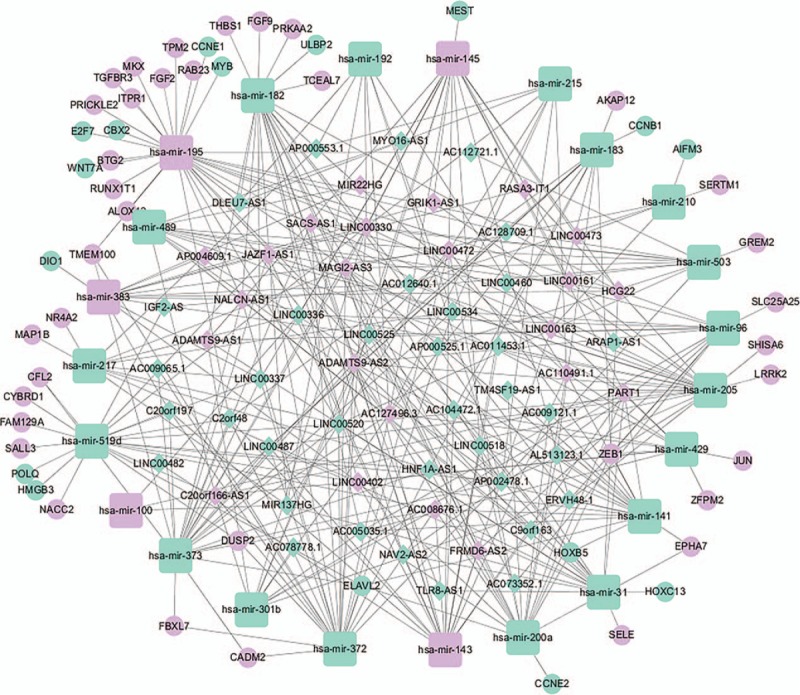
A competitive endogenous RNA network describing lncRNA–miRNA–mRNA interactions in human bladder cancer (BCa). Blue represents up-regulated genes and purple represents down-regulated genes. Diamonds represent lncRNAs, ellipses represent mRNAs, and rounded rectangles represent miRNAs.

To reveal how lncRNA may mediate transcription in BCa by affecting mRNA and miRNA binding, a ceRNA network based on the lists of DElncRNAs, DEmiRNAs, and DEmRNAs was constructed and visualized using Cytoscape software. As shown in Fig. 2, the lncRNA–miRNA–mRNA network was comprised of 23 miRNA nodes, 52 mRNA nodes, 59 lncRNA nodes, and 365 edges. We found that most DEmRNAs were tumor-related genes and including *CBX2*, *DUSP2*, *ELAVL2*, *HOXB5*, and *ZEB1*. Gene information was retrieved from the Onco database (http://www.bushmanlab.org/links/genelist).

### 3.3 Predicated functions of mRNAs within the lncRNA–miRNA–mRNA network

**Figure 3:**
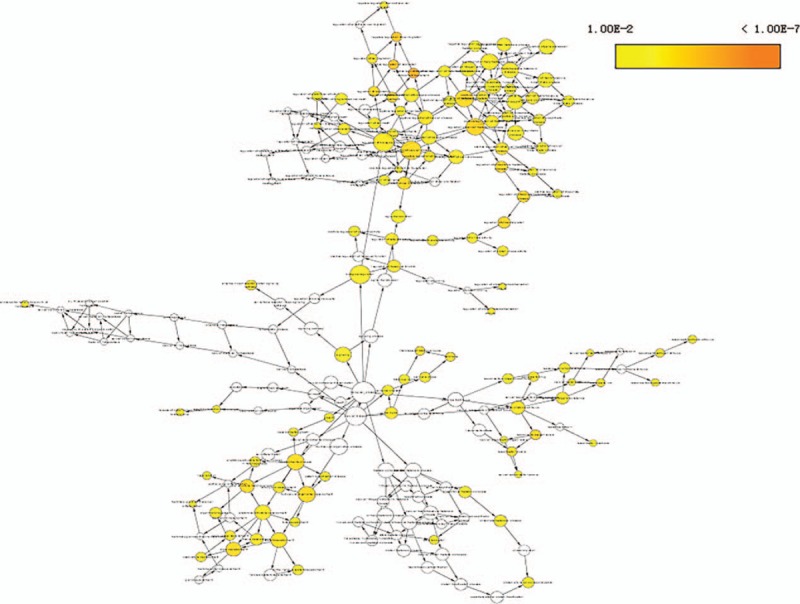
GO terms displayed as an interaction network using the BinGO plug-in for Cytoscape. Yellow nodes are those with *P* < .05 and a Benjamini corrected *P* < .05. GO = gene ontology.

**Figure 4:**
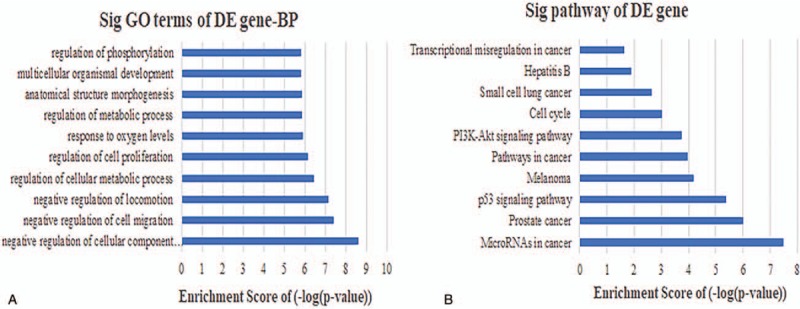
Biological function and pathway analysis of differentially expressed mRNAs. (A) The 10 most significantly enriched GO biological process. (B) The 10 most significantly enriched KEGG pathways. GO = gene ontology.

To establish context of our ceRNA network, we inferred the roles of each lncRNA based on the functions of connected mRNAs. lncRNAs were typically central and connected to one or more mRNAs in the network. The results of an initial GO analysis revealed 300 enriched GO terms at the “Biological Process” level for mRNAs connected to lncRNAs in the network. These included various BP, such as negative regulation of cell migration, regulation of cellular metabolic process, and regulation of cell proliferation. In order to fully explore the inner relationships between these GO terms, a GO interaction network was constructed using the BinGO plug-in for Cytoscape (Fig. 3). We next filtered GO terms with *P* < .05 and Benjamini corrected *P* < .05, leaving 122 that were determined to be significantly enriched. The 10 most significant BP are shown in Fig. 4A. Finally, KEGG pathway analysis revealed that 15 pathways were significantly enriched, particularly microRNAs involved in cancer, prostate cancer, and p53 signaling. The 10 most significant KEGG pathways are shown in Fig. 4B.

### 3.4 The construction of lncRNA–miRNA–mRNA subnetworks containing key lncRNAs

**Figure 5:**
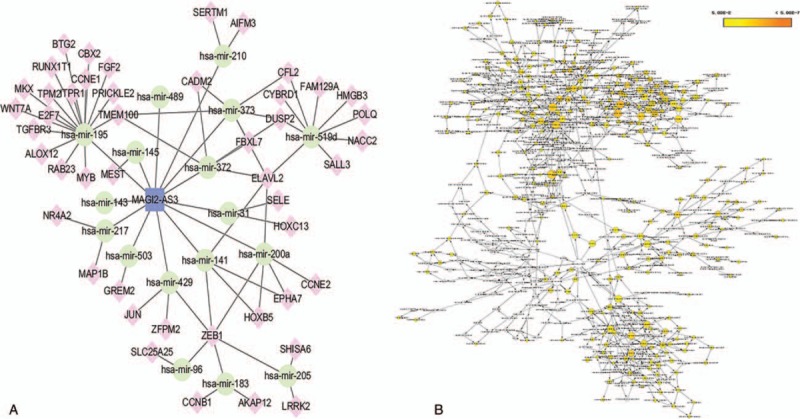
The subnetwork of lncRNA MAGI2-AS3 and related GO terms. (A) The lncRNA MAGI2-AS3 subnetwork. Rounded rectangles represent lncRNAs, ellipses represent miRNA, and diamonds represent mRNAs. (B) GO terms are displayed as an interaction network using the BinGO plug-in for Cytoscape. Yellow nodes are those with *P* < .05 and a Benjamini corrected *P* < .05. GO = gene ontology.

**Figure 6:**
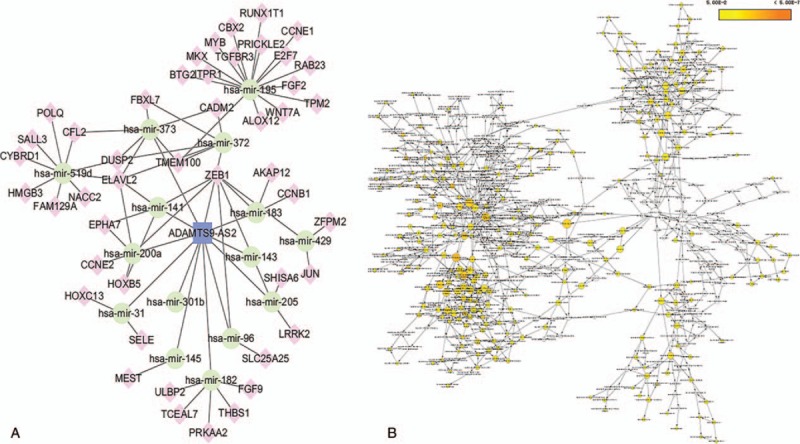
The subnetwork of lncRNA ADAMTS9-AS2 and related GO terms. (A) The lncRNA ADAMTS9-AS2 subnetwork. Rounded rectangles represent lncRNA, ellipses represent miRNA, and diamonds represent mRNAs. (B) GO terms are displayed as an interaction network using the BinGO plug-in for Cytoscape. Yellow nodes are those with *P* < .05 and a Benjamini corrected *P* < .05. GO = gene ontology.

**Figure 7:**
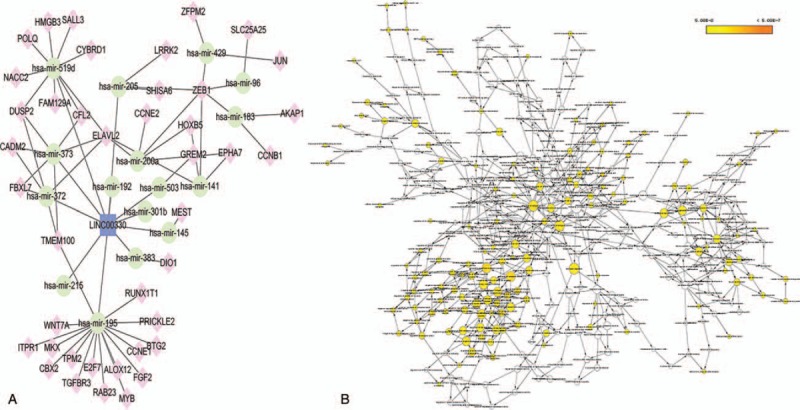
The subnetwork of lncRNA LINC00330 and related GO terms. (A) The lncRNA LINC00330 subnetwork. Rounded rectangles represent lncRNAs, ellipses represent miRNAs, and diamonds represent mRNAs. (B) GO terms are displayed as an interaction network using the BinGO plug-in for Cytoscape. Yellow nodes are those with *P* < .05 and a Benjamini corrected *P* < .05. GO = gene ontology.

**Figure 8:**
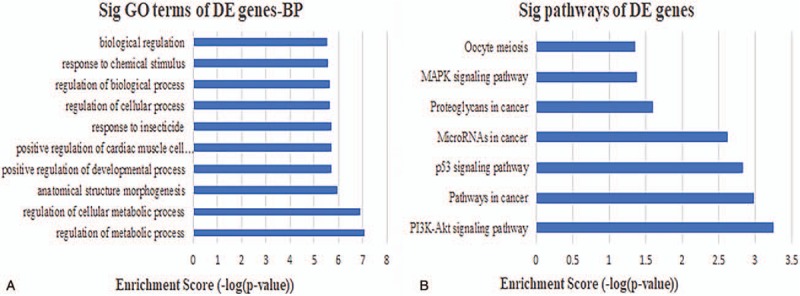
Biological function and pathway analysis of lncRNA MAGI2-AS3-related mRNAs. (A) The 10 most significant mRNA GO biological processes associated with lncRNA MAGI2-AS3. (B) The 10 most significant KEGG pathways in mRNAs linked to lncRNA MAGI2-AS3. GO = gene ontology.

**Figure 9:**
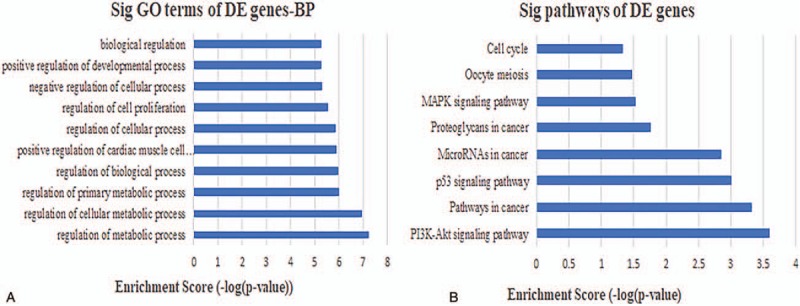
Biological function and pathway analysis of lncRNA ADAMTS9-AS2-related mRNAs. (A) The 10 most significant mRNA GO biological processes associated with lncRNA ADAMTS9-AS2. (B) The 10 most significant KEGG pathways in mRNAs linked to lncRNA ADAMTS9-AS2. GO = gene ontology.

**Figure 10:**
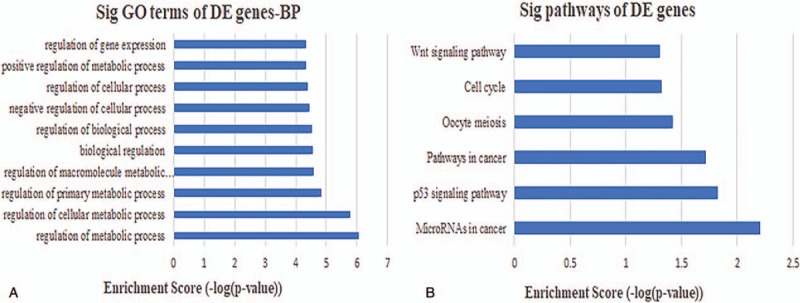
Biological function and pathway analysis of lncRNA LINC00330-related mRNAs. (A) The 10 most significant mRNA GO biological processes associated with lncRNA LINC00330. (B) The 10 most significant KEGG pathways in mRNAs linked to lncRNA LINC00330. GO = gene ontology.

It has been shown in many settings that certain hub nodes play critical roles in biological networks. Accordingly, our ceRNA network revealed 22 nodes with a degree score exceeding 10, indicating that they were particularly important. These hubs consisted of 3 *lncRNAs* and 19 miRNAs (Table 1). The 3 lncRNAs (*MAGI2-AS3*, *ADAMTS9-AS2*, and *LINC00330*) had particularly high node degrees and were likely key nodes involved in the origin and development of BCa. As it has also been demonstrated that lncRNAs, miRNAs, and mRNAs interact and share similar coexpression patterns in ceRNA networks, linked mRNAs and miRNAs were extracted and new subnetworks for these 3 lncRNAs were constructed. As shown in Figs. 5A, 6A, and 7A, the lncRNA *MAGI2-AS3*–miRNA–mRNA subnetwork was comprised of 1 lncRNA node, 17 miRNA nodes, 46 mRNA nodes, and 77 edges. The lncRNA *ADAMTS9-AS2*–miRNA–mRNA subnetwork was composed of 1 lncRNA node, 15 miRNA nodes, 46 mRNA nodes, and 76 edges. The lncRNA *LINC00330*–miRNA–mRNA subnetwork was composed of 1 lncRNA node, 16 miRNA nodes, 41 mRNA nodes, and 70 edges. GO and pathway analysis using these new subnetworks revealed 319 GO terms and 7 pathways enriched in the lncRNA *MAGI2-AS3* network, and 277 GO terms and 8 pathways enriched in the lncRNA *ADAMTS9-AS2* network, and 152 GO terms and 6 pathways enriched in the lncRNA *LINC00330* network. The GO interaction networks are shown in Figs. 5B, 6B, and 7B. The 10 most significant GO terms for each subnetwork are shown in Figs. 8A, 9A, and 10A, and the 10 most enriched KEGG pathways are shown in Figs. 8B, 9B, and 10B.

**Table 1:**
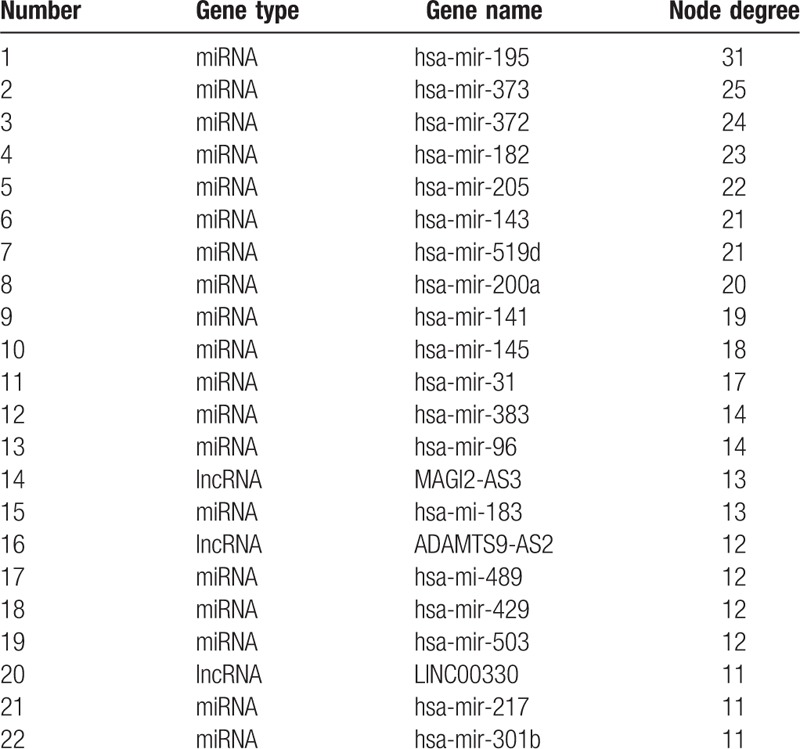
Differentially expressed genes with node degree >10.

### 3.5 Survival analysis

**Figure 11:**
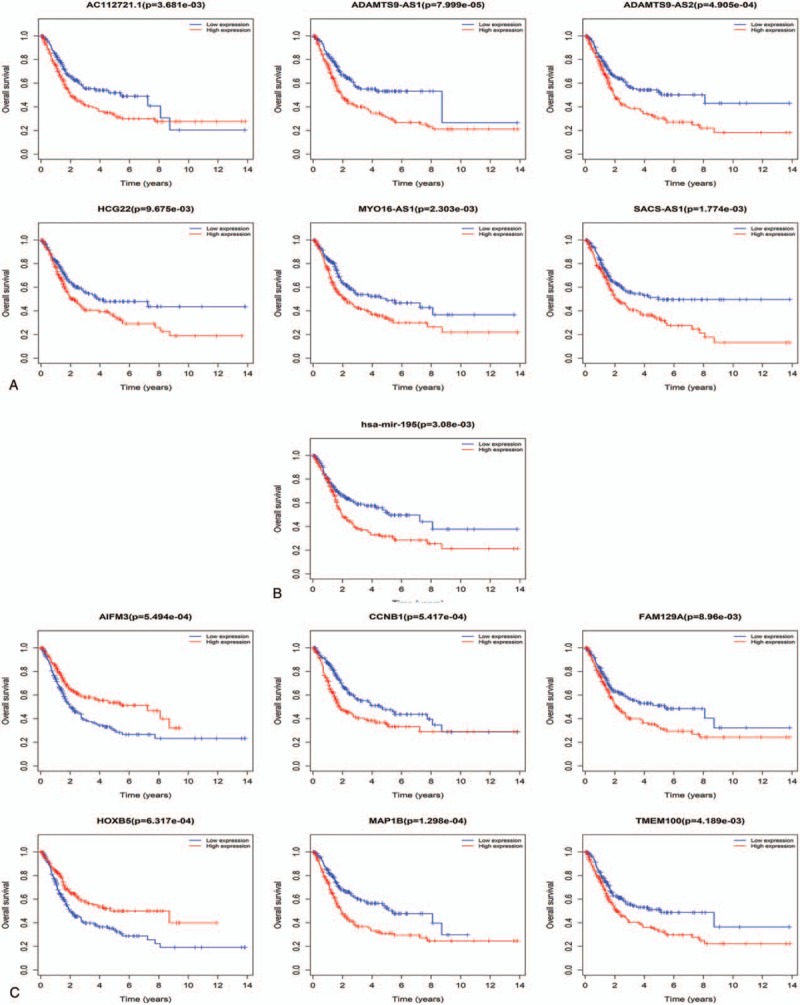
Kaplan–Meier survival curves for differentially expressed RNAs. (A) Six lncRNAs, (B) 1 miRNA, and (C) 6 mRNAs associated with overall survival. The horizontal axis shows overall survival time in years and the vertical axis represents the survival function.

To investigate if any highlighted RNAs affected overall survival in patients with BCa, we performed Kaplan–Meier curve analysis to identify any prognostic signatures. This suggested that all of the key DElncRNAs, except *LINC00330*, associated with BCa progression. High expression of 6 DElncRNAs, including *AC112721.1*, *ADAMTS9-AS1*, *ADAMTS9-AS2*, *HCG22*, *MYO16-AS1*, and *SACS-AS1*, associated with poor prognoses (Fig. 11). Similar to the overall DElncRNA assessments, survival analysis for DEmiRNA-195 showed that DEmirRNAs have negative effects on the overall survival of patients (Fig. 11). In addition, there were 6 DEmRNAs that significantly associated with overall survival, including *CCNB1*, *FAM129A*, *MAP1B*, and *TMEM100*. High expression of these mRNAs negatively associated with overall survival. Conversely, higher expression of *AIFM3* and *HOXB5* predicted greater patient survival time.

## 4 Discussion

Human bladder carcinoma is a considerable global health concern,^[43]^ although the molecular mechanisms that contribute to BCa progression remain unclear. This presents an obstacle to the development of new tools for diagnosis, prognosis, and identifying drug targets. While, ncRNAs have been shown to play a role in tumorigenesis through “miRNA-bridges,” a full understand of the interactions that lead to cancer development are uncertain.^[44,45]^ One tool that can be used investigate the links between ncRNAs and cancer are competing endogenous RNA (ceRNA) networks. These can be applied to establish the post-transcriptional layers of gene regulation in a system. To ascertain whether there are ncRNAs that may be involved in the progression of BCa, we systematically constructed ceRNA networks using patient BCa data and examined links to DElncRNAs. This involved integrating multilevel molecular profiles from large-scale studies found in the TCGA database. The initial ceRNA network we constructed was comprised of 23 miRNA nodes, 52 mRNA nodes, 59 lncRNA nodes, and 365 edges, suggesting numerous lncRNAs that may be involved in gene regulatory networks and BCa genesis. To establish a mechanistic underpinning of the role of lncRNAs in BCa, we used GO and pathway analysis to assess biological functions enriched among the DEmRNAs linked in the ceRNA network. This revealed 300 GO terms enriched in BCa versus normal tissue, 122 of which had *P* < .05 and Benjamini corrected *P* < .05. These significant GO terms included processes involved in the negative regulation of cell migration, regulation of cellular metabolic process, and regulation of cell proliferation, consistent with previous findings.^[46,47]^ Pathway analysis revealed 15 pathways that were enriched in the dataset, primarily involving microRNAs in cancer, prostate cancer, and the p53 signaling. These pathways have all been shown to play important roles in BCa.^[48,49]^

Of the 3 RNA families examined in our study, lncRNAs are considered to have the greatest potential as diagnostic and prognostic biomarkers due to close associations between lncRNAs expression and function.^[50]^ For example, many studies have shown that differential lncRNA expression is closely related to tumor pathogenesis and prognosis in breast cancer, gastric cancer, liver cancer, lung cancer, and kidney cancer.^[51–55]^ However, the diagnostic role of lncRNAs in BCa has not been fully investigated. To establish this, our study used the newly constructed ceRNA network to identify lncRNA hub nodes that could serve as novel biomarkers for BCa clinical diagnosis and treatment. This revealed 3 lncRNAs (*MAGI2-AS3*, *ADAMTS9-AS2*, and *LINC00330*) as topological key nodes with node degrees higher than other lncRNAs. This strongly suggests that these key lncRNAs are involved in BCa genesis and progression, although this will require experiment validation in future studies. Nevertheless, we selected these lncRNAs for further analysis, constructing separate subnetworks centered around the individual lncRNAs.

The lncRNA *MAGI2-AS3* had the highest node degree among the 3 key lncRNAs and was connected to numerous mRNAs and miRNAs that have been previously been shown to be involved in BCa, such as *miR-200* and *miR-143*, validating our approach. Using the lncRNA *MAGI2-AS3–*miRNA–mRNA subnetwork formulated in the study, we speculated that *MAGI2-AS3* may play a role in altering the expression of BCa-related downstream mRNAs through competitive interactions with certain miRNA families, including *miR-200* and *miR-143*. In support of this hypothesis, recent studies have confirmed that *miR-200* and *miR-143* play a crucial role in the development of BCa. For example, Adam et al reported that *miR-200* expression regulates epithelial-to-mesenchymal transition in BCa cell.^[56]^ Further analysis of the GO and pathway analysis for connected mRNAs indicated 319 GO terms and 7 pathways enriched in the network, all of which have been shown to be involved in BCa.^[57]^ In particular, the PI3K/AKT signaling pathway has been shown to trigger a signaling cascade that regulates cancer cell proliferation, invasion, metastasis, and survival, in addition to affecting patient prognosis.^[58–60]^ The PI3K/AKT signaling pathway has also been implicated in BCa and down-regulation of *miR-29c* inhibits cell proliferation in a BCa cell line via the PI3K/AKT pathway.^[61]^

*ADAMTS9-AS2* was the second key lncRNA we identified and is an antisense overlapping lncRNA located upstream from *ADAMTS9*, a newly described tumor suppressor gene.^[18]^ Yao et al examined experimental data to suggest that a decrease in lncRNA *ADAMTS9-AS2* expression associated with the diagnosis, clinicopathological characteristics, and prognosis of glioma. This suggests that *ADAMTS9-AS2* is involved in suppressing cell migration, partly by regulating the protein coding gene *ADAMTS9*.^[62]^ Our survival analysis also indicated that *ADAMTS9-AS2* was important to the progression of BCa and high expression associated with poor prognosis. Examination of the lncRNA *ADAMTS9-AS2*–miRNA–mRNA subnetwork created in the study indicated that *ADAMTS9-AS2* regulates the expression of several BCa-related mRNAs, again through competitive interactions with various miRNAs, such as *miR-200* and *miR-141*. Several studies have confirmed that both *miR-200* and *miR-141* have the potential to be used for the diagnosis of invasive bladder tumors, even after being missed through pathologic assessments of bladder biopsy specimens.^[63,64]^ Our analysis also revealed 277 GO terms and 8 pathways enriched in the mRNAs in the subnetwork, all previously implicated in BCa. Among these signaling pathways, the p53 signaling pathway has been shown to play a critical role in cancer pathogenesis and treatment (apoptosis) resistance. p53 is therefore an important cellular drug target^[65,66]^ and is also involved in the pathogenic mechanisms of BCa. For example, Zhu et al confirmed that downregulation of the tumor suppressor gene *LOC572558* regulates p53 signaling in BCa.^[67]^

The lncRNA *LINC00330* that was highlighted in the study is a relatively underreported lncRNA, although examination of the *LINC00330*–miRNA–mRNA subnetwork suggests it may have the greatest potential for use as a diagnostic or prognostic biomarker. Our analysis suggests that *LINC00330* regulates the expression of numerous BCa-related mRNAs through interactions with miRNAs involved in the regulation of tumor progression, including *miR-195* and *miR-145*. Both *miR-195* and *miR-145* are well-described tumor-suppressing miRNAs that promote apoptosis and inhibit cell proliferation, tumor angiogenesis, and metastasis.^[68–72]^ Our survival analysis indicated that a high expression of *miR-195* correlates with worse prognoses for patients with BCa. Previous reports also suggest that the lncRNA *UCA1* promotes mitochondrial function during BCa by modulating the *miR-195*/ARL2 signaling pathway.^[73]^ We also examined the GO and pathway annotations for the mRNAs in the subnetwork, revealing 152 GO terms and 6 pathways that were enriched. These included numerous signaling pathways, including the Wnt signaling pathway that has been shown to regulate cell fate decisions and to affect cell proliferation, morphology, migration, apoptosis, or differentiation in a wide varieties of tissues.^[74,75]^ The Wnt signaling pathway has also been shown to be involved in BCa. For instance, Chen et al^[76]^ confirmed that *HBO1* promotes BCa proliferation and tumorigenicity via activation of the Wnt signaling pathway. Moreover, we noted that some of the lncRNAs were associated with overall survival in patients with BCa, specifically *AC112721.1*, *ADAMTS9-AS1*, *HCG22*, *MYO16-AS1*, and *SACS-AS1*. However, these key lncRNAs were novel RNAs associated with BCa, suggesting that they are seldom reported.

MRNAs, the hub elements of the ceRNA network, can be directly targeted by miRNAs or have indirect interactions with lncRNAs mediated by miRNAs. Similar to the lncRNAs and miRNAs, some mRNAs were also found to be associated with the survival of BCa patients. Examples of such are mRNA *CCNB1*, *FAM129A*, *MAP1B*, *TMEM100*, *AIFM3*, and *HOXB5*. Chai et al^[77]^ revealed that the high-level expression of CCNB1 is closely associated with poor prognosis in hepatocellular carcinoma (HCC) patients. Taylor et al^[78]^ suggested that low expression of FAM129A acted as predictor of poor prognosis with multiple probes, while low tissue mRNA expression of FAM129A was also associated with a poor outcome.^[79]^ Previous studies have demonstrated that MAP1B interacts with p53 to influence mediated cell apoptosis and proliferation, and loss of this gene may contribute to the cancer cell's ability proliferate.^[80]^ In addition, Han et al had found that TMEM100 was used as cancer suppressor in HCC and nonsmall cell lung cancer.^[81]^ Additionally, AIFM3 has been reported to act as a direct target of miR-210 that is related to proliferation of human hepatoma cells.^[82]^ Moreover, Luo et al have confirmed that HOXB5 was over-expressed in BCa tissues and promoted cell proliferation and the migration of BCa cells, indicating HOXB5s ability to act as an oncogene.^[83]^

## 5 Conclusions

We have constructed an lncRNA–miRNA–mRNA network based on the ceRNA hypothesis. This has enabled us to conduct an overarching review and analysis of lncRNAs that associate with the development of BCa. Three key lncRNAs were identified (*MAGI2-AS3*, *ADAMTS9-AS2*, and *LINC00330*) that likely play important roles in the development and progression of BCa. This study contributes to understanding the pathogenesis of BCa and the role that lncRNAs play in disease progression. These novel lncRNAs may serve as candidate diagnostic biomarkers or therapeutic targets to improve future treatment options for BCa.

## Author contributions

**Conceptualization:** Yuanhao Wu, Geng Li, Guiyun Ma, Jinxin Liu.

**Data curation:** Yuanhao Wu.

**Formal analysis:** Geng Li.

**Methodology:** Jinxin Liu.

**Project administration:** Bin Chen.

**Resources:** Youxin Song.

**Software:** Guiyun Ma, Youxin Song, Wenjia Zhao.

**Supervision:** Guiyun Ma, Wenjia Zhao.

**Visualization:** Youxin Song.

**Writing – original draft:** Naiqiang Zhu, Jingyi Hou.

## Supplementary Material

Supplemental Digital Content

## Supplementary Material

Supplemental Digital Content

## Supplementary Material

Supplemental Digital Content
